# A liquid biopsy signature predicts treatment response to fluoropyrimidine plus platinum therapy in patients with metastatic or unresectable gastric cancer: implications for precision oncology

**DOI:** 10.1186/s12943-021-01483-8

**Published:** 2022-01-03

**Authors:** In-Seob Lee, Zhongxu Zhu, Jeeyun Lee, Joon Oh Park, Xiwei Wu, Tiffany Ong, Sierra Min Li, Xin Wang, Joseph Chao, Ajay Goel

**Affiliations:** 1 Department of Molecular Diagnostics and Experimental Therapeutics, Beckman Research Institute of City of Hope, Biomedical Research Center, Comprehensive Cancer Center, 1218 S. Fifth Avenue, Monrovia, CA 91016 USA; 2 Department of Surgery, Asan Medical Center, University of Ulsan College of Medicine, Seoul, South Korea; 3 Department of Biomedical Sciences, City University of Hong Kong, Hong Kong, China; 4 Division of Hematology-Oncology, Department of Medicine, Samsung Medical Center, Sungkyunkwan University School of Medicine, Seoul, South Korea; 5 Integrative Genomics Core, Beckman Research Institute of City of Hope, Duarte, CA USA; 6 Department of Medical Oncology and Therapeutics Research, City of Hope Comprehensive Cancer Center, 1500 E. Duarte Road, Duarte, CA 91010 USA; 7 City of Hope Comprehensive Cancer Center, Duarte, CA USA

Patients with initially metastatic and/or locally advanced but unresectable gastric cancer (mGC) typically receive systemic chemotherapy based on a combination of fluoropyrimidine and platinum but prognosis in this group remains unsatisfactory [1–6]. Failure to proper transition of patients to subsequent therapies after disease progression on first-line treatment can risk exposing patients to worsening performance status, an upsurge of tumor burden, and exacerbation of toxicity due to the ineffective therapy. Therefore, pre-treatment prediction of response to first-line chemotherapy is highly important for guiding patients to an optimal therapeutic approach and avoiding unnecessary toxicity.

Recent clinical analyses have consistently demonstrated that chemotherapy efficacy is associated with molecular subtyping of gastric cancer (GC) [7, 8]. These data are of significant value as they suggest that optimal chemotherapy should be varied depending on tumor biologic characteristics. However, repeated access to obtain adequate tumor specimens for molecular profiling is limited by the invasiveness of endoscopic or percutaneous biopsy procedures and issues of tumor heterogeneity. Collectively, these observations highlight the unmet need for developing a blood-based biomarker to predict chemo-responsiveness in patients with mGC.

Herein, we conducted systematic, genome-wide expression profiling using small RNA sequencing in serum specimens from patients diagnosed with mGC, followed by bioinformatic approaches to identify a panel of miRNAs that could robustly discriminate responders from non-responders treated with a fluoropyrimidine and platinum doublet regimen. Then, we developed a response-prediction model, which we successfully validated in an independent clinical cohort. This systematic and comprehensive biomarker discovery and validation effort enabled us to identify patients who would benefit from standard chemotherapy, which may lead to more precise clinical decision-making strategies and improve treatment outcomes for patients with GC.

## Results and discussion

### Genome-wide expression profiling identifies a novel 9-miRNA panel for predicting response to systemic chemotherapy

**Fig. 1:**
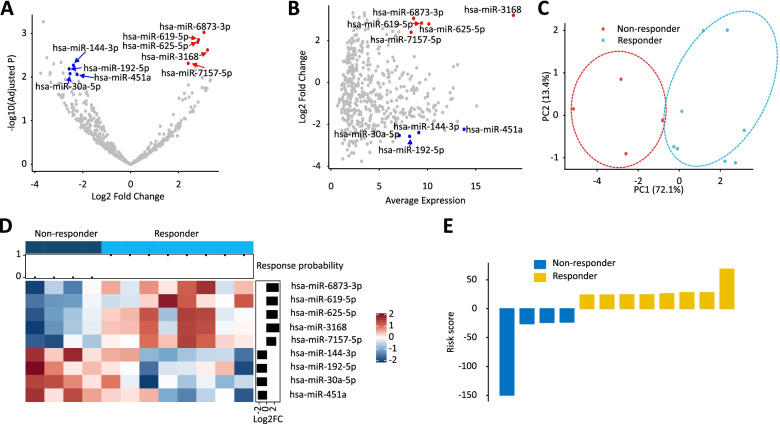
A 9-miRNA panel discovered using small RNA sequencing data identifies patients responsive to systemic chemotherapy. **A** A volcano plot illustrates 9 miRNAs discovered from several bioinformatic approaches in sera from 12 patients with mGC. Among 21 miRNA candidates that were differentially expressed in responders and non-responders, logistic regression and average expression analyses reduced them to a 9-miRNA panel. **B** A volcano plot depicts differential expression of all 9 miRNAs after average expression analysis; 5 candidates were upregulated (red) and 4 were downregulated (blue) in responders vs, non-responders. **C** A principal component analysis shows separate clusters for responder and non-responder groups. **D** A heatmap illustrates clustering of miRNA expression based on response to systemic chemotherapy and estimated probability of chemo-responsiveness from the logistic regression analysis. **E** A waterfall plot demonstrates the ability of the 9-miRNA panel to discriminate responders from non-responders

We analyzed sequencing-based miRNA expression profiling data from patients with mGC. Blood samples were prospectively collected from patients visiting the City of Hope Comprehensive Cancer Center, Duarte, CA, US before the commencement of chemotherapy. The data were from 8 responders (time to progression [TTP] greater than 6 months) and 4 non-responders (TTP less than 6 months), all of whom were treated with fluorouracil, oxaliplatin, and folinic acid (FOLFOX). Among 530 candidate miRNAs (after exclusion of miRNAs with a mean CPM [counts per million] less than 1), we identified 21 miRNAs that were differentially expressed between responders vs. non-responders (*P* < 0.01; absolute log2 fold change > 2). Thereafter, we used an average expression > 7 and an area under curve (AUC) > 0.8 for individual miRNA candidates that discriminated responders from non-responders to identify a panel of 9 miRNAs: miR-30a-5p, miR-144-3p, miR-192-5p, miR-451a, miR-619-5p, miR-625-5p, miR-3168, miR-6873-3p, and miR-7157-5p. All 9 miRNAs showed high statistical significance (Fig. 1A); five miRNAs were upregulated and four were downregulated in the responder group. All miRNAs were accompanied by high average expression after differential expression analysis (Fig. 1B). Principal component analysis illustrated separate clusters for the responder and non-responder groups (Fig. 1C).

Next, we developed a logistic regression model to predict chemo-responsiveness in patients with mGC, which yielded an AUC value of 1.00. Using a response probability score derived from the logistic regression model, we developed a heatmap of the cluster analysis for miRNA expression in relation to treatment response, which showed a separated clustering of expression between the two groups (Fig. 1D**)**. The ability of the miRNA panel to identify the responder group is illustrated in the waterfall plot in Fig. 1E. Taken together, our biomarker discovery effort utilizing rigorous bioinformatic modules allowed us to successfully identify a 9-miRNA panel for prediction of chemo-responsiveness in patients with mGC.

### Clinical validation leads to development of a formula for predicting response to first-line chemotherapy

We next validated the 9-miRNA biomarker panel and developed a response-prediction formula by applying an optimized 2-miRNA biomarker panel consisting of miR-30a-5p and miR-192-5p to a clinical cohort of 29 patients with mGC treated with capecitabine and oxaliplatin (CapeOX) at Samsung Medical Center, Seoul, Korea. Response rate was evaluated using the Response Evaluation Criteria in Solid Tumors (RECIST) criteria version 1.1 [9].

There were 15 responders (1 case of complete response and 14 cases of partial response) and 14 non-responders (10 cases of stable disease and 4 cases of progressive disease). Their median age was 58 years and there were 17 males and 12 females. Median progression-free survival (PFS) and overall survival (OS) for this cohort were 4.1 and 16.1 months, respectively. There was no difference in gender, tumor location, Lauren classification, type of second-line treatment, or mean OS between responders and non-responders, but mean PFS was significantly longer in the responder group (9.1 vs. 2.7 months, *P* = 0.007).

**Fig. 2:**
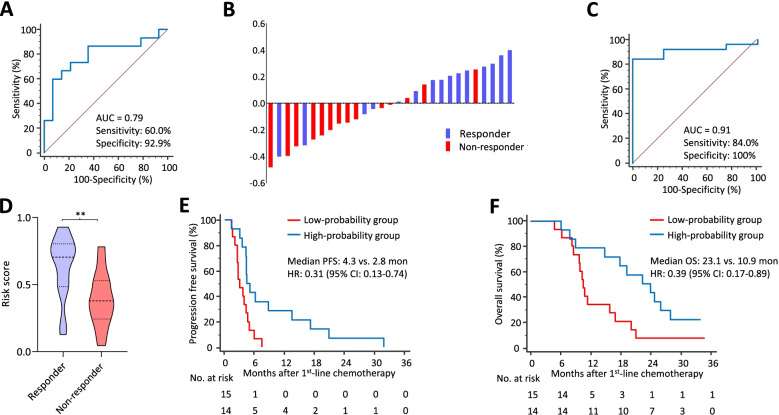
The 2-miRNA biomarker panel predicts chemo-responsiveness and prognosis in patients with mGC. **A** A ROC curve shows the accuracy of the 2-miRNA biomarker panel for predicting responsiveness to systemic chemotherapy (responder group vs. non-responder group). **B** A waterfall plot illustrates the ability of the biomarker panel to identify responders. **C** A ROC curve shows the ability of the 2-miRNA biomarker panel to successfully identify patients with progressive disease on first-line chemotherapy. **D** A response-probability score derived from the biomarker model was significantly different between responder vs. non-responder groups (** *P* < 0.01). **E** A Kaplan-Meier curve indicates a significant difference in median progression-free survival (PFS) between patients grouped as having a high- vs. low-probability for response to chemotherapy, determined by the cutoff values derived from the response-prediction model. **E** A Kaplan-Meier curve indicates a significant difference in overall survival (OS) between the two groups

For biomarker validation, we performed RT-qPCR assays for each of the 9 miRNAs to measure expression in all samples. From these data, we developed a 2-miRNA-based prediction formula using logistic regression analysis through a backward elimination method, which exhibited an AUC of 0.79 in discriminating responders from non-responders (95% CI: 0.60–0.92, sensitivity 60.0%, specificity 92.9%, *P* = 0.021; Fig. 2A). A waterfall plot illustrates the ability of our 2-miRNA biomarker panel to identify responders (Fig. 2B).

Next, we assessed the ability of the biomarker panel to identify only patients with progressive disease, in whom treatment intensification or altered chemotherapeutic drugs should be considered instead of doublet regimen. Our biomarker panel successfully identified patients who were expected to have the worst prognosis, with a corresponding AUC of 0.91 (95% CI: 0.74–0.98, sensitivity: 84.0%, specificity: 100%, *P* = 0.008; Fig. 2C). We also verified that the response score was significantly different between two groups (*P* = 0.006) (Fig. 2D).

### The 2-miRNA biomarker panel robustly stratifies prognosis of patients with mGC

We dichotomized patients into groups with high- or low-probability of chemo-responsiveness, according to cutoff threshold values derived from the identical 2-miRNA prediction model and the proportion of responders was significantly higher in high-probability group than low-probability group (78.6% vs. 26.7%, *P* = 0.006). Then we performed Kaplan-Meier analyses to determine the prognostic significance of our biomarkers. PFS in patients with a high probability of chemo-responsiveness was significantly better compared to PFS in a low probability group (median PFS: 4.3 vs. 2.8 months, *P* = 0.009), with a hazard ratio of 0.31 (95% CI: 0.13–0.74, *P* = 0.008) (Fig. 2E). We also observed a significant difference in OS between two patient groups (median OS: 23.1 vs. 10.9 months, *P* = 0.021), with a hazard ratio of 0.39 (95% CI: 0.17–0.89, *P* = 0.025) (Fig. 2F). Other clinical variables were not associated with prognosis. These results highlight that, in addition to its ability to predict chemo-responsiveness, our biomarkers are clinically significant in stratifying the prognosis of patients with mGC.

### Expression of the two miRNAs is significantly upregulated upon tumor progression

To determine whether expression of the two miRNAs changed following tumor progression, we analyzed and compared serum samples collected before initiation of first-line chemotherapy vs. upon disease progression in the clinical cohort. Upon tumor progression, we observed significant upregulation of both miR-30a-5p and miR-192-5p (*P* < 0.001).

## Conclusions

Through a systematic and comprehensive discovery and validation effort, we developed a novel 2-miRNA biomarker panel that predicts chemo-responsiveness to fluoropyrimidine plus oxaliplatin therapy in patients with mGC. Our biomarkers may ultimately facilitate individualized treatment and mediate alternative cytotoxic chemotherapy strategies to improve survival outcomes in patients suffering from this malignancy.

## Data Availability

All data are available within the article.

## Abbreviations

AUC: Area under the curve
CI: Confidence interval
CPM: Counts per million
GC: Gastric cancer
mGC: Metastatic or unresectable gastric cancer
OS: Overall survival
PFS: Progression-free survival
RT-qPCR: Real-time quantitative reverse transcription polymerase chain reaction
TTP: Time to progression
